# Preparation of a Novel Nanocomposite and Its Antibacterial Effectiveness against *Enterococcus faecalis*—An In Vitro Evaluation

**DOI:** 10.3390/polym14081499

**Published:** 2022-04-07

**Authors:** Jerry Jose, Kavalipurapu Venkata Teja, Krishnamachari Janani, Mohammad Khursheed Alam, Osama Khattak, Mahmoud Gamal Salloum, Shilpa S. Magar, Shaliputra P. Magar, Shanmugam Rajeshkumar, Ajitha Palanivelu, Kumar Chandan Srivastava, Deepti Shrivastava

**Affiliations:** 1 Department of Conservative Dentistry & Endodontics, Saveetha Dental College & Hospitals, Saveetha Institute of Medical & Technical Sciences, Saveetha University, Chennai 600077, Tamil Nadu, India; jerryjosekavungal@gmail.com (J.J.); drajitharijesh@gmail.com (A.P.); 2 Department of Conservative Dentistry and Endodontics, Mamata Institute of Dental Sciences, Bachupally, Hyderabad 500090, Telangana State, India; metejaendo@gmail.com; 3 Department of Conservative Dentistry and Endodontics, SRM Dental College, SRM Institute of Science and Technology, Chennai 600089, Tamil Nadu, India; jananik6@srmist.edu.in; 4 Orthodontics, Department of Preventive Dentistry, College of Dentistry, Jouf University, Sakaka 72345, Saudi Arabia; mkalam@ju.edu.sa; 5 Department of Public Health, Faculty of Allied Health Sciences, Daffodil International University, Dhaka 1207, Bangladesh; 6 Department of Restorative Dentistry, College of Dentistry, Jouf University, Sakaka 72345, Saudi Arabia; dr.osama.khattak@jodent.org (O.K.); dr.shilpa.magar@jodent.org (S.S.M.); 7 Prosthetic Dental Science Department, College of Dentistry, Jouf University, Sakaka 72345, Saudi Arabia; mjsalloum@ju.edu.sa; 8 Oral Medicine & Radiology, Department of Oral & Maxillofacial Surgery & Diagnostic Sciences, College of Dentistry, Jouf University, Sakaka 72345, Saudi Arabia; shaliomdr@gmail.com; 9 Nanobiomedicine Lab, Department of Pharmacology, Saveetha Dental College & Hospitals, Saveetha Institute of Medical & Technical Sciences, Saveetha University, Chennai 600077, Tamil Nadu, India; ssrajeshkumar@hotmail.com; 10 Department of Periodontics and Preventive Dentistry, College of Dentistry, Jouf University, Sakaka 72345, Saudi Arabia

**Keywords:** dental restorations, resin composites, multifunctional nanoparticles, microbial sensitivity tests, *Enterococcus faecalis*

## Abstract

The interest in the use of green-mediated synthesis of nanoparticles (NPs) is shown to have increased due to their biocompatibility and reduction of overall production costs. The current study aimed to evaluate a novel nanocomposite (NC) prepared by using a combination of zinc oxide, silver and chitosan with lemon extract as a cross-linking agent and assessed its antimicrobial effectiveness against *Enterococcus faecalis* (*E. faecalis*). The NPs and NC were prepared individually using a modification of previously established methods. Ananalys is of the physiochemical properties of the NC was conducted using ultraviolet-visible spectroscopy (UV-Vis) (Shimadzu Corporation, Kyoto, Japan). and transmission electron microscopy (TEM) imaging(HR-TEM; JEOL Ltd., Akishima-shi, Japan. The microbial reduction with this novel NC was evaluated by measuring the minimum inhibitory concentration (MIC) and minimum bactericidal concentration (MBC) using a tube assay analytic technique. A time-kill assay analysis was conducted to evaluate the kinetic potential against *E. faecalis* at different time intervals. The novel NC showed a homogenous nanoparticle size under TEM imaging and under UV-Vis established an absorption range of 350–420 nm making it similar to its individual counterparts. The MIC and MIB were measured at 62.5 ± 20 mg/L (*p* < 0.05) and 250 ± 72 mg/L (*p* < 0.05), respectively. A time-kill assay analysis for the NC showed 5 h was required to eradicate *E. faecalis*. Based on the achieved results, it was seen that the novel NC using a combination of silver, zinc oxide and chitosan showed improved antimicrobial action against *E. faecalis* compared with its individual components under laboratory conditions. A complete eradication of 10^8^ log units of *E. faecalis* at 250 mg/L occurred after a total of 5 h. These preliminary results establish the use of lemon extract-mediated silver, zinc and chitosan-based NC had an antibacterial effectiveness against *E. faecalis* similar to the individual counterparts used for its production under laboratory conditions.

## 1. Introduction

The use of nanotechnology in the medicinal field has been a boon in this present technological era due to its numerous versatile applications [1]. This is mainly due to its particle size being as small as 1 to 100 µm giving it a physiochemical characteristic which enables it to be incorporated for medicinal purposes [2]. Due to this property, nanoparticles (NP) have been shown to possess a profound ability to be used in nanoscales, enabling them to be used in various applications and giving them not only a better mechanical property advantage but also better antimicrobial action [3].

The use of NPs as an adjuvant has been discussed for use in dental restoration and it has been established that they improve characteristic properties; for instance, NPs incorporated into dental restorative materials were seen to increase antibacterial effectiveness to a certain extent [4]. Recent research has emphasized incorporating different NPs for increasing the bond strength and as tools for caries inhibition [5]. They are also known to decrease dental plaque biofilm viability and formation as well as to promote remineralization [6]. The incorporation of nanoparticles has established a role in increasing aesthetic properties. For instance, titanium oxide when incorporated into nanoparticles has a significant role in increasing the aesthetic characteristic features of composite-based restoration material [7].

*Enterococci* have been shown to cause infection in humans in various organs, such as in the urinary tract, endocardium and burn wounds. *Enterococcus faecalis* (*E. faecalis*) plays a profound role in root canal failures and ranges from being 4 to 40% [8] of primary endodontic infections. Since it possesses the ability to reside in the deeper layer of dentinal tubules, it canbe exempt from the chemicals used for disinfection of the canals to a certain extent [9]. The virulence of *Enterococci* can be described as their ability to survive, acquire, accommodate and encode virulence traits to enable themselves to withstand adverse environments. The toxins released by these organisms can cause tissue damage leading to increased mortality and systemic toxicity in certain scenarios [10]. Current evidence suggests that *E. faecalis* is considered to be one of the primary pathogens in endodontic infections and has specific characteristics in order to retain within the root canal system in spite of the chemo-mechanical procedures employed [11].

Antimicrobial action for different NPs is shown to be composed of varied mechanisms [12]. The most commonly used NPs discussed that possess antimicrobial effects are silver (Ag), copper (Cu) and chitosan (CS). For instance, they are able to interact with peptidoglycan-composed cell walls causing cell lysis by increasing reactive oxygen species (ROS) generation. They can also prevent DNA replication, interact with bacterial proteins and prevent the synthesis of proteins [13,14]. The major proposed mechanism of action seems to be contact-mediated bactericidal action. This action is carried out by a positively charged interaction with negatively charged bacterial membranes allowing the diffusion and dissolution of the bacterial cell membrane components [15]. Conventional synthesis of NPs has many applications and the use of synthetic reducing agents such as sodium borohydride and ethylene glycol procures the NPs at a faster rate. It is also shown to absorb harsh chemicals during procurement on the surface that canfurther progress to toxicity issues [16].

Considering all the prior mentioned factors, this current study focused on synthesizing a novel nanocomposite (NC) based on the combination of Ag, Zn and CS with lemon extract as a crosslinking agent. The Zn-, Ag- and CS-based NPs and the novel NC were subjected to characterization using UV-vis and TEM. Clinical isolates of *E. faecalis* were used to assess the antimicrobial action of NPs and NC by using an agar well diffusion method. The MIC and MBC of the novel NC were analyzed using a tube assay analytic technique. Furthermore, to determine the kinetic effect, a time-kill assay analytical technique was employed to analyze the rate of action of the novel NC against *E. faecalis*.

## 2. Materials and Methods

### 2.1. Characteristics of This Study

The experimental in vitro study was conducted in the research laboratory of the university with the protocol approved by the university (SRB/SDC/ENDO-1802/21/03).

### 2.2. Materials

Fresh lemons were purchased from the local market in Chennai and washed under distilled water to remove any surface impurities. Each lemon was cut individually and was squeezed to release the juice. The obtained juice was centrifuged at 10,000 rpm for 10 min. The resultant suspension was filtered by 0.45 µm micropore membrane filter (Sigma-Aldrich, St. Louis, MO, USA) followed by 0.2 µm Millipore membrane filter (Sigma-Aldrich, St. Louis, MO, USA). Ag, Zn and CS used for the experiment were purchased from Sigma-Aldrich Company, St. Louis, MO, USA. This extract was used as the cross-linking agent for synthesis of the NPs and NC as described below.

### 2.3. Synthesis of the Various NPs

The synthesis of the Zn-, Ag- and CS-based NPs and NC was performed according to the methodology given by Nishibuchi et al. [17]. The prior procured lemon extract was put in a 500 mL beaker followed by the synthesis of NPs and NC which was conducted as follows:

#### 2.3.1. Synthesis of Lemon Extract Mediated Zinc NP (Li-ZnNP)

A total of 25 mL of the prior-mentioned lemon extract was taken and added to 75 mL of distilled water in an Erlenmeyer flask at a controlled room temperature with continuous trituration in a mechanical titrator. This was followed by the addition of Zn(NO_3_)_2_ solution periodically for a 60 min timeframe with continuous trituration. A yellowish cream-colored precipitate was formed classically indicating the formation of zinc hydroxide. The reaction mixture was allowed to be left idle for 30 min for complete reduction of zinc hydroxide and a centrifugation at 10,000 rpm for 10 min was conducted once the final reduction of clear fluid was achieved. The resultant mixture was then heated to 400 °C for 1 h which finally formed a fine solid mass. This solid mass was grounded mechanically using a mortar and pestle until a fine powder form was obtained. To maintain purity, the achieved powder was filtered through 100 µm nylon.

#### 2.3.2. Synthesis of Lemon Extract-Mediated Silver NP (Li-Ag NP)

A total of 10 mL of the prior-mentioned lemon extract was taken and added to 100 mL of distilled water in an Erlenmeyer flask in a dark chamber at room temperature and 100 mL of 0.01 N aqueous AgNO_3_ was added periodically with constant trituration at a controlled temperature of 30 °C for 60 min. The resultant solution underwent a colour change to yellowish brown in colour indicating the formation of AgNP. The stability of this achieved NP was determined by storing at 4°C and checking periodically. Later, the solution centrifuging at 10,000 rpm for 10 min and the pellet were re-dispersed in Millipore water (Sigma-Aldrich, St. Louis, MO, USA) to remove any unreacted molecules. This centrifugation process was repeated thrice and pellets were freeze dried in a lyophilizer (Modulyo 230 freeze dryer, Thermo Electron Corp., Waltham, MA, USA). The purified dried powder was used as the NPs and was filtered through 100 µm nylon mesh to maintain purity.

#### 2.3.3. Synthesis of Lemon Extract-Mediated Chitosan NP (Li-CSNP)

A total of 10 mL of lemon extract was taken and added to 100 mL of distilled water in an Erlenmeyer flask at room temperature followed by 50 mL of 0.75% (*w*/*v*) chitosan dissolved in 1% acetic acid, which was added periodically under continuous trituration in a magnetic stirrer of 700 rpm under room temperature for 60 min. The supernatant liquid was discarded and the achieved particles were freeze dried. The final preparation was then grounded to fine powder form and filtered through a nylon mesh of 100 µm to maintain purity.

### 2.4. Synthesis of Lemon Extract-Mediated Silver/Zinc/Chitosan-Based Nanocomposite (Li-Ag/Zn/CS NC)

A total of 10 mL of lemon extract was added to 50 mL of distilled water placed in an Erlenmeyer flask and subjected to trituration in a magnetic stirrer for 12 h. A total of 50 mL of AgNO_3_, 50 mL of ZnNO_3_ and 50 mL of CS was added subsequently at intervals of 60 min each until a homogenous suspension was achieved. The supernatant liquid was discarded and particles were freeze dried. The final preparation was then grounded to fine powder form and filtered through a nylon mesh of 100 µm to maintain purity.

### 2.5. Characterization of the Various Nanoparticles (NPs)

The crystallinity and phases of the various NPs were evaluated using UV-vis spectroscopy and TEM imaging. An aqueous colloidal solution was used for each NP as the agent for characterization and UV-Vis spectrometry was used operating at an optical length cuvette of 50 mm. The absorbance spectra of the nanoparticles were analysed by using a ‘SHIMADZU’ UV 1800 spectrophotometer (Shimadzu Corporation, Kyoto, Japan). The characterization was conducted at a wavelength of around 200–800 nm followed by the SPR wavelength (ƛSPR) of each element being evaluated. Furthermore, the concentration of each nanoparticle was determined using the same technique. The TEM images were taken using a JEOL JEM-2100 high-resolution transmission electron microscope (HR-TEM; JEOL Ltd., Akishima-shi, Japan). The TEM images were used to analyse the shape and size of the nanoparticles and the nanocomposite. Samples for the TEM studies were prepared by placing a drop of the aqueous suspension of particles on carbon-coated copper grids followed by solvent evaporation under vacuum conditions and were subjected to further analysis. The polydispersity of the NPs and NC was assessed based on the values and examination results achieved using UV-Vis and TEM imaging.

### 2.6. Antimicrobial Activity of the Different Nanoparticles against Enterococcus faecalis

The agar well diffusion method was carried out to determine antimicrobial susceptibility against *E. faecalis*. The plates were designed for dispensing using isolates of *E. faecalis* (ATCC 29212) which were spread evenly over the Petridish followed by a sterile agar medium which was uniformly poured in each corresponding well of 6 mm diameter using a sterile well borer. The dishes were incubated overnight at 37 °C in different concentrations, from 25, 50, 75 to 150 µg/L. The diameter of each well was subsequently measured and the zone of inhibition was compared individually.

The MIC and MBC were conducted using the CLSI 2015 guidelines for different nanoparticles against *E. faecalis*. They were evaluated using a microbroth dilution technique in which different titres of solution were taken for assessment [18].

### 2.7. Time-Kill Assay

A bacterial concentration of between 6 and 8 log colony-forming units (CFU)/mL was used in the time-kill experiments. Test tubes containing Mueller–Hinton broth using 1, 2 or 4× MIC or without antibiotic were inoculated with overnight cultures of *E. faecalis*. The cultures were then incubated in a shaker (Julabo, Allentown, PA, USA) at 37 °C for 2 h, 4 h, 6 h and 24 h. At the end of each time period, ten-fold serial dilutions were prepared with PBS and 100 L samples were plated onto Mueller–Hinton agar plates in triplicate. The effect of antibiotic carryover of all three was minimized by the PBS dilution of the samples. The CFU for each strain at different time points were counted after 18 h. Plates with 30–300 colonies were used for CFU counts. The time-kill assay was analysed against *E. faecalis* at hourly time intervals from 1 to 5 h.

### 2.8. Statistical Analysis

The statistical analysis was carried out using SPSS v21.0 (IBM Corp., Armonk, NY, USA). The normality test analysis was conducted using Kolmogorov–Smirnov and Shapiro–Wilk tests and showed a non-parametric distribution. The data were analysed using Friedman’s test to assess the comparison of absorbance at different time intervals 530 nm between the treatment groups, and pairwise comparisons of value were conducted at different time intervals.

## 3. Results

### 3.1. Characterization of the Nanoparticles (NPs)

**Figure 1:**
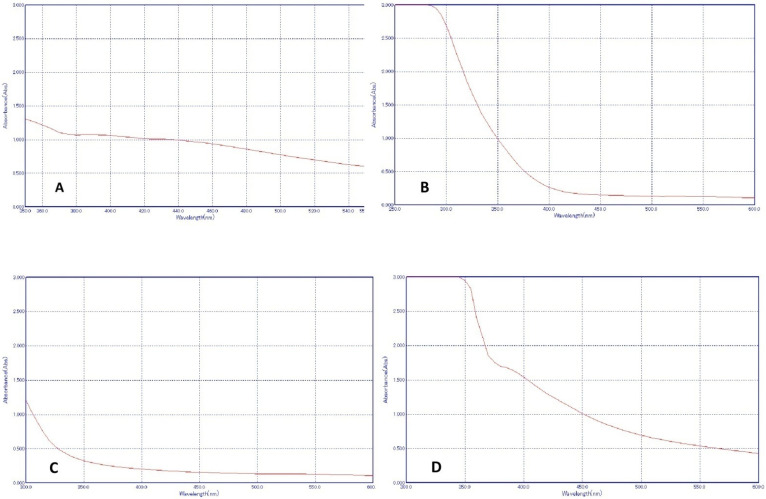
UV-Vis spectroscopy analysis of Li-AgNP (**A**), Li-ZnNP (**B**), Li-CSNP (**C**) and Li-Ag/Zn/CSNC (**D**) (from left to right).

The characterization of the NPs was performed using UV-Vis and TEM. Figure 1 shows the absorbance rate of each NP was recorded; Li-Ag NP showed an absorbance range of 420–460 nm. Li-Zn NP was shown to have a range of 380–450 nm while Li-CS NP showed an absorbance range of 390–420 nm. The novel Li-Ag/Zn/CS NC showed an absorbance range of 370–420 nm.

**Figure 2:**
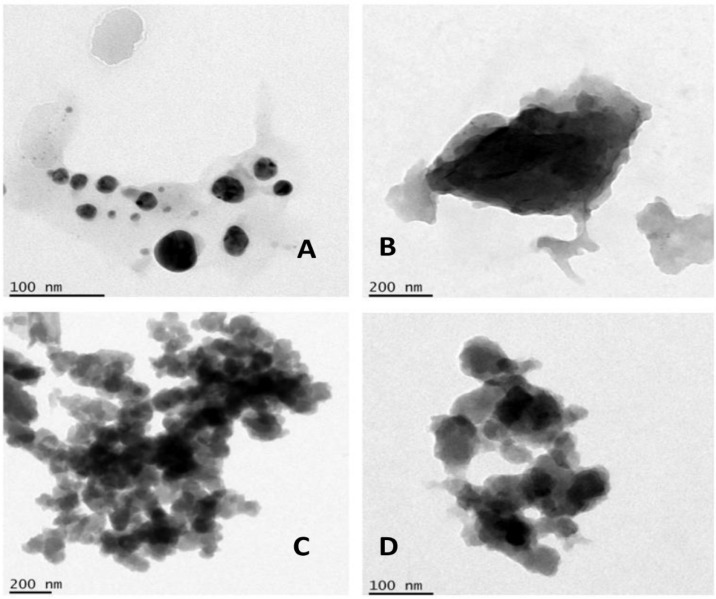
Transmission electron microscopy imaging of Li-AgNP (**A**), Li-ZnNP (**B**), Li-CSNP (**C**) and Li-Ag/Zn/CSNC (**D**) (from left to right).

Figure 2 denotes the TEM imaging of the NPs and NC showing spherical agglomerate formation for Li-AgNP. Li-ZnNP showed an agglomerate mass which could not be distinguished easily. Li-CSNP showed agglomerate formation in clusters and Li-Ag/Zn/CSNC showed an agglomerate formation with spatial distribution.

### 3.2. Antimicrobial Susceptibility

The antimicrobial susceptibility was assessed at different concentrations: 25 µg/L, 50 µg/L, 75 µg/L, 100 µg/L and 150 µg/L. The results of the agar well diffusion test are shown in Table 1. The antimicrobial activity was shown to exhibit better at 25 µg/L, 50 µg/L, 75 µg/L and 100 µg/L for Li-AgNP and the novel Li-Ag/Zn/CSNC showed similar levels to other assessed NPs. Table 2 denotes the MIC value of Li-Ag/Zn/CSNC which was valued at 62.5 mg/L at different time intervals using the Friedman’s test. Table 3 denotes an intragroup comparison of Li-Ag/Zn/CSNC at different time intervals. Significant differences were seen at1 h, 3 h, 2 h and 5 h, respectively.

### 3.3. Antibacterial Activity

**Figure 3:**
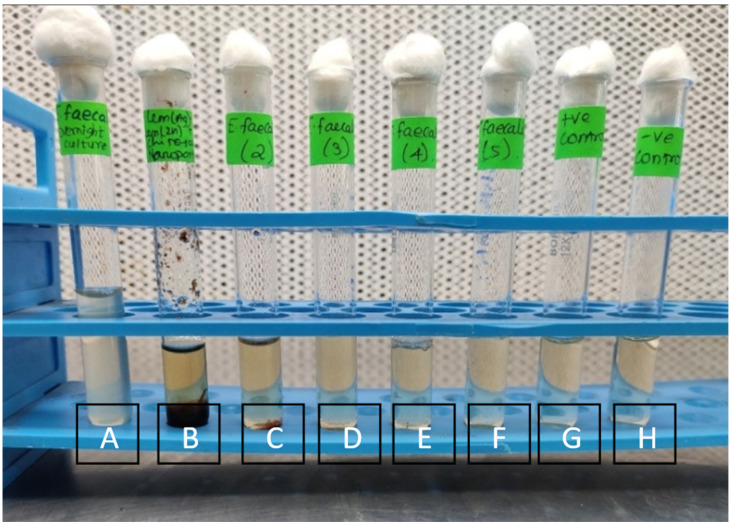
Minimum inhibitory concentration determination of the Li-Ag/Zn/CSNC against *Enterococcus faecalis* biofilm using tube assay analytical technique (A—chitosan nanoparticle, B—silver, zinc, chitosan nanoparticle, C–F—*E. faecalis*, G—positive control, H—negative control).

**Figure 4:**
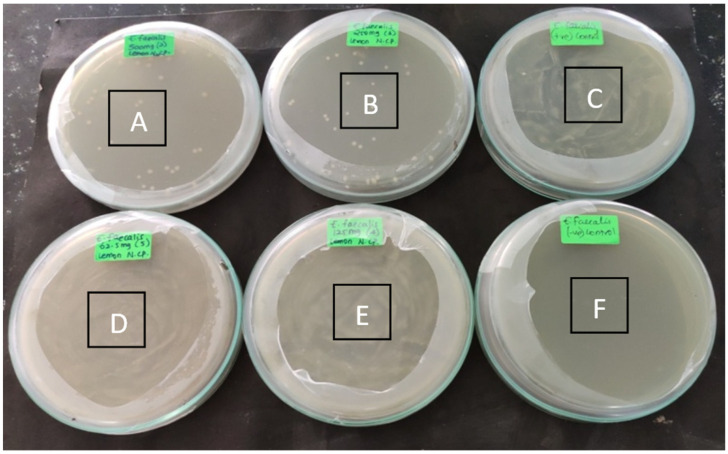
Minimum bacterial concentration of the Li-Ag/Zn/CSNC against *Enterococcus faecalis* biofilm. The minimum bacterial concentration was seen at 250 mg/mL (A—*E. faecalis* 500 mg, B—*E. faecalis* 250 mg, C—positive control, D—*E. faecalis* 62.5 mg, E—*E. faecalis* 12.5 mg, F—negative control).

The MIC and MBC were analyzed during which the cultures were analyzed at different concentrations. The MIC of Li-Ag/Zn/CSNC were seen to be mediated highest at 62.5 mg/mL (*p* < 0.05) (Figure 3) and the MBC at 250 mg/mL (*p* < 0.05) (Figure 4).

### 3.4. Time-Kill Assay

**Figure 5:**
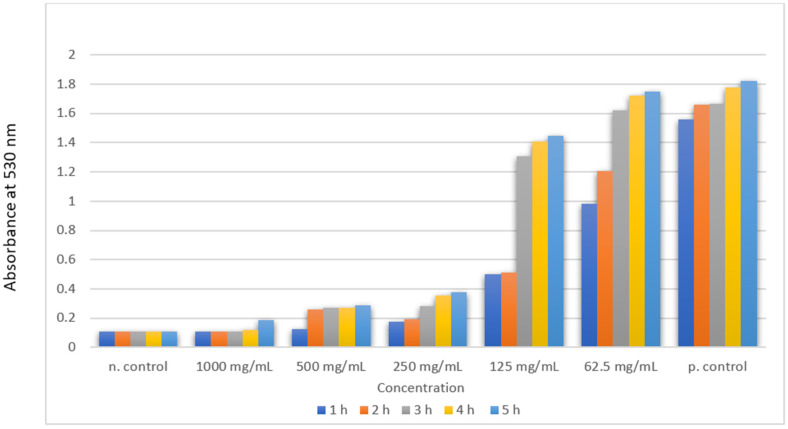
Time-kill kinetic assay from 1 h to 5 h of Li-Ag/Zn/CSNC against *E. faecalis* biofilm.

The time-kill assay was conducted in a timeframe of 1 to 5 h. Figure 5 denotes the time-kill kinetics of the Li-Ag/Zn/CSNC against *E. faecalis*. The absorption was set at 530 nm. Sequential reduction of the *E. faecalis* count was seen from 1 to 4 h. At 250 mg/mL, complete antimicrobial reduction was seen at 5 h with the negative control showing complete inhibition and the positive control showing higher microbial action compared to the NC counterpart.

## 4. Discussion

*E. faecalis* are common members of the healthy intestinal flora and are shown to be extremely virulent in nature. This virulence is achieved by the release of the toxin cytolysin and the presence of the surface protein Esp. [19]. The presence of Esp. as a surface protein is shown to have a determinant role in the pathogenicity of *E. faecalis*, helping it to produce biofilms [20].

Various studies [21,22,23] have used NPs as antimicrobial agents for applications. They have a great advantage due to their small particulate size and potential to dissociate into smaller structures where conventionally sized particles would not be able to reach. With regard to the oral environment, NPs have been shown to possess a positive effect in the reduction of oral biofilms and microorganisms and have shown individual antimicrobial effect as well as effect in combination with various materials to serve a variety of purposes, which influenced our study in the selection of the particular NPs.

AgNP has shown to have greater interest among various researchers and has been tried individually and in several combinations to obtain an increased antimicrobial effect [24]. The antimicrobial mechanisms of AgNP have been explained in two forms, one being its ability to release ROS which inhibit the respiratory enzyme and attack the cell itself and the other being its ability to act on DNA peptides causing cell death to occur [25]. Other greatly studied components are CS and Zn, with the former being an adeacetylated derivative of chitin with a contact-mediated kill, altering cell wall permeability causing lysis to occur, and the latter showing a similar mode of action which penetrates into bacterial cells and causes cell lysis to occur [26,27]. Most of these NPs have proved to have an excellent antimicrobial activity against various microorganisms, such as *E. coli*, *S. aureus* and *E. faecalis* [28]. Similarly, they have been shown to have ability to be incorporated into materials. For instance, ZnNP has been shown to incorporate well in materials such as gelatin films and even in already developed material components such as intracanal medicaments, increasing the dynamics of antimicrobial activity against various microorganisms [29,30,31].

In endodontics, the importance of the coronal seal and its influence on the outcome of endodontic therapy has been greatly emphasized. It was emphasized by Gillen et al. [32] that the quality of endodontic treatment was greatly enhanced by root canal filling and adequate restoration. It has been reported that the biomaterials used currently for coronal sealing have significant limitations, such as shrinkage, dissolution in oral environment and sensitivity to moisture. The novel NC procured exhibited similar properties to NPs and had the potential to be incorporated into these materials, which could greatly enhance the properties of the coronal seal and allow perfect quality sealing with minimal leakage [33].

In case of a pulpal and periapical infection, it has been shown that biofilms have some degree of resistance to conventional antimicrobial treatment due to the presence of an efflux pump (AccrAB) giving them increased resistance to antimicrobial agents [34]. Due to this factor, conventional disinfectants in endodontics can exhibit reduced potential especially against *E. faecalis*. This organism has been shown to have a high virulence factor and releases cytolysin and other bacteriocins which have an ability to suppress the growth of other organisms [35].

Green synthesis of NPs is an underused method which has been shown to possess advantages such as less cytotoxicity and being more cost effective as well as being able to produce metal ions faster when compared to other methods [36]. Lemon obtained from a plant source is shown to have many bioactive components such as flavonoids, essential oils which give lemons antioxidative, anti-inflammatory and antimicrobial activities and are devoid of any adverse reactions [37]. Lemon extract was taken as a reducing agent in our study since it had shown the ability to bind components easily as well as consisting of a high number of polyphenols giving it good antimicrobial action as well [38]. The function of across-linking agent is primarily to link the NPs together such that they act as a single unit. For this study, lemon extract was the binder which enabled binding at the functional site of Ag, Zn and CS, enabling them to form a stable structure [39]. The combined action of Li-Ag/Zn/CSNC showed a better antimicrobial effect negating the disadvantages of the individual NPs showing a combined effect. The results of this study were found to be in accordance with a previous published study which stated that the combination of Ag-, Zn- and CS-based NPs showed effective inhibition of bacterial and fungal growth [40]. Although AgNP is shown to have excellent antimicrobial efficacy, it is known to exhibit a certain amount of toxicity towards cells [41]. Moreover, studies have shown that silver-containing materials tend to cause discoloration [42].Although AgNP is shown to have excellent antimicrobial efficacy it is known to show toxicity towards cells [41]. The combined effect of the NPs in our study would be able to negate all these effects and give a synergistic effect to increase antimicrobial action, though this is the subject of a later study approach.

The study was conducted in accordance with the CLSI guidelines of 2015. MIC and MBC are some of the tests used to assess the antibacterial effects of certain materials. The MIC method shows a serial dilution concentration as it determines the lowest concentration and antimicrobial properties. In the present study, it showed a minimum concentration of 62.5 µg/mL. In a study by Hernández-Sierra et al. [43], it was seen that the MIC and MBC results of Ag and Zn-based NPs were an MIC of 4.86 ± 2.71 μg/mL and an MBC of 6.25 μg/mL; for Zn, the MIC was 500 ± 306.18 μg/mL and the MBC was 500 μg/mL against *S. mutans* which shows an increased concentration is required to achieve an MIC and MBC. This shows that in combination the NP has an increased effect compared with its individual counterparts. The MBC of our novel NC was seen to be at 250 µg/mL. Panpaliya et al. [44] performed an invitro evaluation to assess the MIC and MBC of AgNP against various microbes and found it to have a maximum MBC of 52 µg/mL.

Previous studies have demonstrated that nanocomposites such as 10% ZnNC have the potential for areduction of biofilm [45] and also have a reported 50% decrease in the turbidity of resuspended biofilms grown on dental composites [46]. Furthermore, it was revealed that bacterial growth in the presence of composites showed a modest suppression of biofilm growth, whereas 1% Ag-containing composite discs inhibited biofilm growth by 10^3^. Additionally, independent of ZnNP, the Ag-based composite discs had a 5 ± 0.5 mm inhibitory zone, which was not seen in any of the other composites [47]. On the contrary, the results of the present study showed ZnNP to possess higher MIC and MBC than AgNP. The results of this study were found to be in accordance with the results of a previously published study which stated composite resin containing ZnNP was superior to AgNP and CSNP and had shown effective inhibition of bacterial growth within 48 h [40].

A time-kill kinetic assay is used to assess the ability to eliminate bacteria in a continuous period of time [48,49,50]. It is considered the most appropriate method for analyzing antibacterial action and gives a dynamic model of the interaction between the antimicrobial agent and microbial strain [51]. Theophel et al. [52] proposed that real-time antimicrobial susceptibility testing will provide much more dynamic information than static assessment, leading to a better understanding of drug interaction with amicrobe at a dynamic level rather than a static point interface. In our study, a time-kill assay study was conducted to eliminate *E. faecalis* in a continuous period, providing detailed information about the antimicrobial action. In our study, the NC was shown to have an effect against *E. faecalis* from 1 h, giving an initial effect at 62.5 mg/L at 1 h and with a maximum antimicrobial effect seen at 250 mg/L concentration with a time frame of 5 h required for the complete disintegration of the *E. faecalis* biofilm, showing much better antimicrobial action than that of its individual components. This can be considered the future basis for further evaluation of the present study.

## 5. Strength Limitations and Future Directions

The current study aimed to procure and analyse a novel NC made from Ag, Zn and CS using lemon extract as cross-linking agent. The study showed its antibacterial effectiveness against *E. faecalis*, a pathogen resistant to various antibacterial substances, and analysed the stability as well as the MIC and MBC in comparison to its individual counterparts. One of the limitations of the current study was not testing the novel NC in an ex vivo environment which could simulate similar conditions to the oral cavity. Further ex-vivo-based studies and clinical trials would determine the antibacterial effectiveness of this NC and practical clinical applications.

## 6. Conclusions

Li-Ag-, Li-Zn- and Li-CS-based NPs had shown sufficient antimicrobial effects against *E. faecalis* biofilm based on the agar well diffusion method. Li-Ag/Zn/CSNC was able to show a much higher antimicrobial action at a higher concentration than its individual counterparts. Further evaluation showed that Li-Ag/Zn/CSNC had an MIC of 62.5 mg/L and an MBC of 250 mg/L. A time-kill assay analysis of Li-Ag/Zn/CSNC was able to show that the NC had the ability to disintegrate the *E. faecalis* biofilm after a 5 h interval. Further studies are required to assess Li-Ag/Zn/CSNC and its potential properties for further application for medicinal purposes.

## Figures and Tables

**Table 1:** The antimicrobial susceptibility of nanocomposite and individual components against *E. faecalis.*.

Zone of Inhibition against *Enterococcus faecalis*					
Sample	Zone of Inhibition (Concentration) in mm				
	25 µg/L	50 µg/L	75 µg/L	100 µg/L	150 µg/L
Li-AgNP	27	30	32	34	40
Li-ZnNP	12	14	23	25	29
Li-CSNP	14	18	25	26	32
Li-Ag/Zn/CSNC	23	24	28	30	42

**Table 2:** Evaluation of the MIC at 62.5 mg/mL was determined for Li-Ag/Zn/CSNC at different time intervals using Friedman’s test.

Time Intervals	Mean ± SD	Mean Ranks	Chi Square	*p* Value
1 h	0.987 ± 0. 006	2.00	12.00	0.017 *
2 h	1.211 ± 0.004	5.00		
3 h	1.623 ± 0.004	11.00		
4 h	1.606 ± 0.006	8.00		
5 h	1.646 ± 0.001	14.00		

* *p* value less than 0.05 is statistically significant.

**Table 3:** Pairwise comparison of MIC was determined for Li-Ag/Zn/CSNC at different time intervals; *p* < 0.05 was considered statistically significant.

Time Intervals	Mean Difference	Chi Square	*p* Value
1 h vs. 2 h	−0.22367	−0.775	0.439
1 h vs. 3 h	−0.63600	−2.324	0.020 *
1 h vs. 4 h	−0.61933	−1.549	0.121
1 h vs. 5 h	−0.65900	−3.098	0.002 *
2 h vs. 3 h	−0.41233	−1.549	0.121
2 h vs. 4 h	−0.39567	−0.775	0.439
2 h vs. 5 h	−0.43533	−2.324	0.020 *
3 h vs. 4 h	0.01667	0.775	0.439
3 h vs. 5 h	−0.02300	−0.775	0.439
4 h vs. 5 h	−0.03967	−1.549	0.121

* *p* value less than 0.05 is statistically significant.

## Data Availability

The data set used in the current study will be made available on reasonable request.
